# Operationalizing Iterative RE-AIM in two pragmatic nursing home trials to support an adaptation learning cycle

**DOI:** 10.3389/frhs.2026.1847212

**Published:** 2026-09-30

**Authors:** Annie W. Yang, Sophia Geisser, Amy Recker, Valerie Clark, Christine W. Hartmann, A. Lynn Snow

**Affiliations:** 1 National Clinician Scholars Program, Yale School of Medicine, New Haven, CT, United States; 2 VA Connecticut Healthcare System, West Haven, CT, United States; 3 Geriatric Research Education and Clinical Center, James J Peters VA Medical Center, Bronx, NY, United States; 4 Alabama Research Institute on Aging and the Department of Psychology, The University of Alabama, Tuscaloosa, AL, United States; 5 Brown University School of Public Health, Providence, RI, United States; 6 Center for Health Optimization and Implementation Research, VA Bedford Healthcare System, Bedford, MA, United States; 7 Department of Public Health, Zuckerberg College of Health Sciences, University of Massachusetts Lowell, Lowell, MA, United States; 8 Tuscaloosa VA Medical Center, Tuscaloosa, AL, United States

**Keywords:** adaptation, FRAME-IS, Iterative RE-AIM, nursing home, pragmatic trial

## Abstract

**Introduction:**

Complex, heterogeneous settings such as nursing homes require ongoing adaptation of interventions and implementation strategies. Iterative RE-AIM offers a structured approach to guide adaptations, but how the approach is operationalized may need to vary across implementation contexts. We describe how two pragmatic nursing home trials operationalized Iterative RE-AIM with FRAME-IS to enable a systematic adaptation learning cycle.

**Methods:**

We conducted a narrative analysis of two ongoing multi-site trials: TEAM EFFORT (focused on falls reduction in State Veterans Homes) and 40 WINKS (focused on sleep improvement in nursing home residents with dementia). Both used RE-AIM for evaluation and FRAME-IS to document adaptations. Each operationalized the Iterative RE-AIM steps based on study scope, resources, team structure, and implementation timing, with a primary focus on refining a coach-supported implementation strategy.

**Results:**

TEAM EFFORT embedded adaptation-related data collection, deliberation, and decision-making within weekly team meetings, supported by an ongoing input tool; 40 WINKS used individual-level surveys followed by structured, once-per-wave team gatherings to decide on adaptations. These two operationalizations illustrate how the timing, methods, and cadence of an adaptation learning cycle based on Iterative RE-AIM can vary in response to implementation context.

**Discussion:**

Iterative RE-AIM can be operationalized differently across implementation contexts while remaining faithful to its underlying intent. Paired with FRAME-IS, Iterative RE-AIM can enable an adaptation learning cycle that integrates systematic documentation of adaptations with structured team deliberation. Rather than prescribing a single Iterative RE-AIM implementation protocol, these findings suggest that operationalization can respond to the context in which implementation occurs.

## Introduction

A linear approach to implementing interventions often falls short. Many interventions require continual adaptation as they interact with and evolve within complex, real-world environments. Adaptations may involve either the evidence-based intervention itself or the implementation strategies (1–3). We define adaptations as intentional modifications made to the delivery or components of an evidence-informed intervention or implementation strategy to improve fit or effectiveness within a new context (1, 4).

Adaptations are particularly challenging to implement in nursing homes. Nursing homes are complex, heterogeneous environments where “one size fits all” approaches to quality improvement are less likely to succeed than approaches tailored to local needs (5). Frequent organizational change, staffing instability, and evolving regulatory and policy environments create ongoing adaptation needs throughout the implementation journey. These adaptation needs may involve either the intervention itself or the implementation strategy used to support intervention delivery, integration, and sustainment. Iterative RE-AIM has emerged as a strategy to help adaptations develop throughout implementation, however its use in nursing homes is not well described (2, 6, 7).

Iterative RE-AIM addresses limitations in the original RE-AIM framework. The original RE-AIM framework is widely used to plan and evaluate interventions by assessing Reach, Effectiveness, Adoption, Implementation, and Maintenance, but it doesn't offer a natural opportunity to address adaptations mid-implementation. The original RE-AIM provides a valuable framework for evaluating implementation outcomes, but it is most commonly applied (1) before implementation to guide planning and (2) after implementation to evaluate outcomes. In contrast, Iterative RE-AIM was developed by Glasgow and colleagues as a complement to the original framework, extending the use of RE-AIM into the implementation period itself to assess progress toward RE-AIM goals. It adds a structured, repeatable process for systematically responding to adaptations and adaptation needs that arise over time. Thus, the Iterative RE-AIM approach supports ongoing learning and adaptation (6, 7).

Iterative RE-AIM is put into practice through four steps: Identify, Score, Review, and Evaluate. Together, these steps underpin a systematic cycle that supports implementation teams to collectively reflect on adaptation as it unfolds, appraise emerging adaptation needs and observed adaptations, make adaptation decisions, and execute and evaluate those decisions over time. The process is intended to be repeated throughout implementation at a cadence appropriate to the needs of the project (e.g., bimonthly, quarterly). Iterative RE-AIM has been applied to various levels of organization (e.g., hospital, national Ministry of Health), clinical settings (Federally Qualified Healthcare Centers, Veteran's Administration), and intervention types (hypertension medication delivery, colorectal cancer screening) (6). Examples of implementation strategy adaptation include modifying training (in-person to hybrid), making medication pickup more flexible by including family members, and de-implementing less effective strategies. But our research discovered that Iterative RE-AIM could itself be adapted and operationalized.

Our research team has extensive experience partnering with nursing homes to adapt and implement quality improvement interventions (8–10). Using two multi-stage, nursing home-based pragmatic clinical trials, we compare two distinct operationalizations of the Iterative RE-AIM approach to illustrate how implementation teams can remain faithful to its underlying intent while adapting its operationalization to local implementation contexts. These operationalizations demonstrate how Iterative RE-AIM, used together with FRAME-IS, can support systematic cycles of adaptation and learning in complex implementation settings.

### Overview of the two pragmatic trials

The two pragmatic trials evaluated the same evidence-based frontline staff huddling intervention (the LOCK program, described below) while operationalizing the Iterative RE-AIM approach differently to fit their respective implementation contexts. Table 1 summarizes key characteristics of the two trials: (1) “Pragmatic Trial to Increase Quality of Care in State Veterans Homes: Improving Safety Using an Evidence-based, Frontline Staff Huddling Practice” (known from here on by its study acronym of “TEAM EFFORT”) and (2) “Enhancing Sleep Quality for Nursing Home Residents with Dementia: Pragmatic Trial of an Evidence-Based Frontline Huddling Program” (known from here on by its study acronym of “40 WINKS”). At the time of this writing, the two trials were ongoing; this paper describes work completed to date. Although the studies shared the same intervention (“LOCK program”), key research team members (principal investigators and quantitative methods experts), and population of interest (nursing home residents), they differed in important contextual characteristics that shaped how each trial operationalized the Iterative RE-AIM process (3, 5, 11, 12).

**Table 1:** Descriptive overview of TEAM EFFORT and 40 WINKS trials.

Trial characteristic	TEAM EFFORT	40 WINKS
Setting	State Veterans Homes nursing homes	Non-federal or non-state nursing homes, nested within private corporations
Funder	Department of Veterans Affairs, Office of Research and Development	National Institute on Aging
Target clinical area	Falls	Sleep
Participants	Veterans, long-stay residents	Residents with dementia
Intervention	LOCK program	LOCK program
Primary Outcome	Rate of any fall (secondary data)	Resident sleep (primary data)
Primary outcome data	Administrative data (Minimum Data Set) used to measure falls	Primary data collection via Actiwatches to measure sleep and movement
Total number of participating nursing homes	8 State Veterans Homes	28 nursing homes
Total number of study waves	2	4
Intervention period	6 months	9 months
Coaching model	External coaching only	External & internal coaching
Coaching	TEAM EFFORT research team VA specific team coaches Nursing home leadership team (e.g., administrator, social worker, specific to the individual facility; they attend weekly coaching calls)	40 WINKS research team External facilitation team coaches (research employees) Nursing home corporation-specific community coaches (nursing home corporation employees) Nursing home leadership team (e.g., administrator, social worker, specific to the individual facility; they attend weekly coaching calls)

TEAM EFFORT, pragmatic trial to increase quality of care in State Veterans homes: improving safety using an evidence-based, frontline staff huddling practice. 40 WINKS, enhancing sleep quality for nursing home residents with dementia: pragmatic trial of an evidence-based frontline huddling program.

TEAM EFFORT was funded by the Department of Veterans Affairs (VA) Office of Research and Development and focused on reducing falls for residents of participating State Veteran Home nursing homes; 40 WINKS was funded by the National Institute on Aging and focused on improving resident sleep and daytime activity for residents of three community nursing home corporations. The studies also differed in organizational structure, scope, resources, and research team composition. TEAM EFFORT had a smaller budget and scope than 40 WINKS. TEAM EFFORT was conducted by a single integrated VA research team working with one type of nursing home organization, whereas the 40 WINKS research team spanned multiple organizations and partnered with multiple community nursing home corporations. These contextual differences created distinct implementation challenges and opportunities that influenced different operationalizations of the Iterative RE-AIM process.

Both trials paired the Iterative RE-AIM approach with the Framework for Reporting Adaptations and Modifications to Evidence-based Implementation Strategies (FRAME-IS). Building on previous work demonstrating how original RE-AIM can be paired with original FRAME to identify and report modifications to evidence-based practices (11), we paired Iterative RE-AIM with FRAME-IS to support a more systematic adaptation learning process during implementation. FRAME-IS provided a systematic method for tracking, organizing, documenting, and classifying adaptations (3, 11, 12), whereas the Iterative RE-AIM process provided the structured mechanism through which research teams gathered and organized information on adaptations; appraised their significance; reflected on potential responses and made adaptation decisions; and executed and evaluated those decisions over time. Together, FRAME-IS and Iterative RE-AIM enable an adaptation learning cycle that integrates structured team reflection with systematic documentation of adaptations.

### Intervention

#### The LOCK program

The TEAM EFFORT and 40 WINKS studies both implemented the LOCK intervention, an evidence-based frontline staff huddling approach designed to improve quality of care in nursing homes (13–15). The two studies shared the same core LOCK intervention process while tailoring the clinical content to different quality improvement priorities (falls prevention in TEAM EFFORT and sleep improvement in 40 WINKS). The core elements of the LOCK intervention are the following: (1) “Learn from bright spots” (focus on evidence of positive change); (2) “Observe” (collect data through systematic observation); (3) “Collaborate in huddles” (conduct frontline staff huddles); and (4) “Keep it bite-size” (limit activities to 5–15 min). The intervention is grounded in the regular use of brief, interdisciplinary frontline staff huddles that engage all staff members, particularly those providing direct care to residents, in collaborative problem solving and rapid-cycle quality improvement strategies. Further details about the LOCK program can be found elsewhere (13, 14, 16).

### Implementation strategy

Both studies implemented the LOCK intervention using a coach-supported implementation strategy that equipped nursing home leadership teams to implement the LOCK intervention locally. Each study employed coaches (separate from the research team) to train nursing home sites on the intervention. Coaches were individuals from outside the nursing homes who were already well versed in the LOCK program. The research teams provided additional training on LOCK as needed. Coaches served as trainers (external facilitators) for the nursing home leadership and champion teams (internal facilitators). The nursing home leadership and champion teams were nursing home staff (e.g., administrator, director of nursing, activities professional, social worker, nurse manager representatives). Each site's leadership and champion team was coached on how to operationalize and implement the LOCK program at the frontline. The leadership and champion team then worked with frontline, direct-care nursing home staff on implementing the study's LOCK program. The coaching process for the nursing home leadership and champion teams generally included weekly coaching calls (30–50 min), provision of educational materials, and communicating as needed with a coach. Although the core implementation strategy was shared across studies, its operationalization evolved over time through an adaptation learning cycle initiated when either (1) an adaptation need was discovered or (2) an adaptation was observed in practice.

#### Coaching curriculum

To improve consistency while allowing context-responsive implementation, both studies developed structured coaching curricula to guide interactions between coaches and nursing home leadership and champion teams. These curricula defined timelines and educational goals, activities, and milestones to guide coaching interactions. The coaching curricula became one of the principal targets of implementation strategy adaptation throughout both studies. As adaptation needs were identified and adaptations were observed in practice, research teams used the adaptation learning cycle to review, appraise, and refine curriculum content and coaching supports while maintaining the core functions of the implementation strategy.

## Method

### Original RE-AIM framework

Both trials used the original RE-AIM framework to orient the study and guide development of the study's implementation and evaluation plan (Table 2). The framework provided a common structure for defining and assessing Reach, Effectiveness, Adoption, Implementation, and Maintenance outcomes throughout the studies.

**Table 2:** How TEAM EFFORT and 40 WINKS operationalized the Iterative RE-AIM approach to support an adaptation learning cycle.

Iterative* RE-AIM Step^a^	Original Iterative RE-AIM guidance	TEAM EFFORT Operationalization & interpretation of Iterative RE-AIM step	40 WINKS Operationalization & interpretation of Iterative RE-AIM step
Identify (Organize)	Identify Iterative RE-AIM participants and process coordinator (facilitator)	Established the adaptation learning team by identifying coaches and research team members (facilitator was member of research team).	
	Identify/review RE-AIM goals and outcomes	Adaptation learning teams explicitly focused on adaptations. Adaptations naturally converged around intervention modifications and implementation strategy modifications. Because modifications were intended to improve RE-AIM outcomes, discussing intervention and implementation strategy became the practical mechanism to advance RE-AIM domains.	
Score (Appraise)	Distribute survey to each team member to share perceived importance of RE-AIM outcomes and current progress on outcomes	Used an “input tool” to solicit and organize appraisal input from coaches and research team members through weekly team meetings. A formal survey was not distributed. Team members gave appraisals and verbal input during weekly discussions. Input was centered on adaptation needs, observed adaptations, and proposed adaptations to the coaching curriculum.	Administered a survey to coaches and research team members to solicit appraisal input ahead of the once-a-wave evaluation meeting. The survey asked research and coaching team members to evaluate and rate adaptations implemented in the current wave, assess understanding of the coaching curriculum, and propose new adaptations.
Review (Reflect)	Review survey results	Research team reflected on all adaptations and potential solutions together at weekly team meetings, sought input from coaching team, and decided which adaptations to implement in the next week. All of the “Score” and “Review” activities occurred concurrently during a weekly meeting.	Research and coaching team reviewed survey results together during the evaluation meeting. Team members reflected on adaptations implemented in the current wave, proposed adaptations from the survey, brainstormed new adaptations, and prioritized adaptations proposed for the next wave.
	Identify 1−2 RE-AIM dimensions to address Consider strategies or adaptations that might address the desired areas of improvement Decide on timeline to implement action plan		
Evaluate (Learn)	Evaluate success of adaptation and action plans Decide timing of next Iterative RE-AIM round	Evaluated success of adaptations to the coaching curriculum based on RE-AIM components weekly. Next Iterative RE-AIM round timed weekly.	Evaluated success of adaptations to the coaching curriculum based on the RE-AIM components once a wave. Next Iterative RE-AIM round timed for the following wave's evaluation meeting.

FRAME-IS, framework for reporting adaptations and modifications to evidence-based implementation strategies. ^a^The parenthetical terms (Organize, Appraise, Reflect, Learn) represent the research team's interpretation of the underlying intent of each Iterative RE-AIM step. Across both studies, this intent was instantiated in ways consistent with Iterative RE-AIM while adapting specific procedures to local implementation contexts.

“Reach” was assessed by the number of residents who were the focus of huddles. TEAM EFFORT assessed “Effectiveness” by using administrative data records to calculate the rate of falls (primary outcome) and by surveying staff on staff-oriented outcomes (e.g., improvement in communication, problem solving, fall prevention). 40 WINKS assessed “Effectiveness” by measuring resident sleep (primary outcome) and using administrative data records for secondary outcomes (pain, psychotropic medication use). For the “AIM” components, both trials assessed staff adoption, implementation, and maintenance. “Adoption” was measured as staff attendance and participation at huddles. “Implementation” was assessed through competency assessments. Coaches and nursing home leadership and champion teams completed competency assessments that tracked how nursing homes developed LOCK program-related skills. The assessments informed coaches’ plans for subsequent coaching sessions and informed the research teams’ plans to modify the coaching curriculum. “Maintenance” was measured by the sustainment of the program after coaching ended. While the original RE-AIM provided a baseline framework to evaluate the intervention, it assumed the implementation strategy would remain static.

These outcome assessments informed ongoing understanding of trial progress but, consistent with the original RE-AIM framework, did not by themselves provide a structured process for identifying, deliberating about, or responding to emerging adaptation needs during implementation.

### FRAME-IS

As described earlier, the Framework for Reporting Adaptations and Modifications to Evidence-based Implementation Strategies (FRAME-IS) was used in both studies. FRAME-IS has been used in long-term care research to help oversee modifications to implementation strategies (3, 5, 11, 12). FRAME-IS served as the primary mechanism for creating a systematic record of adaptations, their characteristics, and the underlying rationale.

### Operationalization of the Iterative RE-AIM approach

The Iterative RE-AIM approach is organized around four steps: Identify, Score, Review, and Evaluate (17). But we believe these four step names are more narrow than what the steps represent, so we use the *parenthetical* terms below to describe how we interpreted each step’s broader intent.

- Identify: Core functions included identifying the individuals who would participate in the Iterative RE-AIM process, identifying adaptation situations, capturing and organizing adaptation-related information; to describe these core functions, we used the interpretive label *organize*.
- Score: Core functions included surveying, assessing or gauging the importance or significance of adaptation information; to describe these core functions we used the interpretive label, *appraise.*
- Review: Core functions included reflecting on potential responses to adaptation situations and making adaptation decisions; to describe these core functions, we used the interpretive label *reflect.*
- Evaluate: Core functions included executing those adaptation decisions and restarting the cycle with new organizing activities to gather information for evaluation and support continual learning; to describe these core functions, we used the interpretive label *learn*.

Each study instantiated these four steps in ways consistent with the framework's underlying intent rather than implementing the steps as a rigid protocol (Table 2). The timing, specific activities, and operationalization of individual steps differed across trials, reflecting differences in study scope, organizational context, available resources, and implementation timing.

### Iterative RE-AIM, FRAME-IS and the adaptation learning cycle

Because implementation occurred within complex nursing home environments, research teams regularly encountered situations where the existing intervention or implementation strategy approaches no longer fit local needs. In some cases, adaptation needs emerged through structured team reflection. In others, adaptations arose spontaneously in the field: implementation had already changed within participating nursing homes, and research teams had to decide whether to support and standardize, modify, or discourage those changes.

To support team deliberations about adaptations, both studies combined FRAME-IS with the Iterative RE-AIM approach to create an adaptation learning cycle (Figure 1). Within this cycle, research teams gathered and organized information on adaptation situations (organize); appraised their significance (appraise); reflected on potential responses and decided on adaptations as a team (reflect); and executed and evaluated those decisions over time (learn). Iterative RE-AIM and FRAME-IS served complementary functions within this adaptation learning cycle. Iterative RE-AIM provided the structured process by which research teams worked through adaptation-related developments, situations, and needed responses. FRAME-IS was then used to capture and classify adaptations resulting from those deliberations, as well as adaptations emerging from the field. Figure 1 illustrates the adaptation learning cycle.

**Figure 1:**
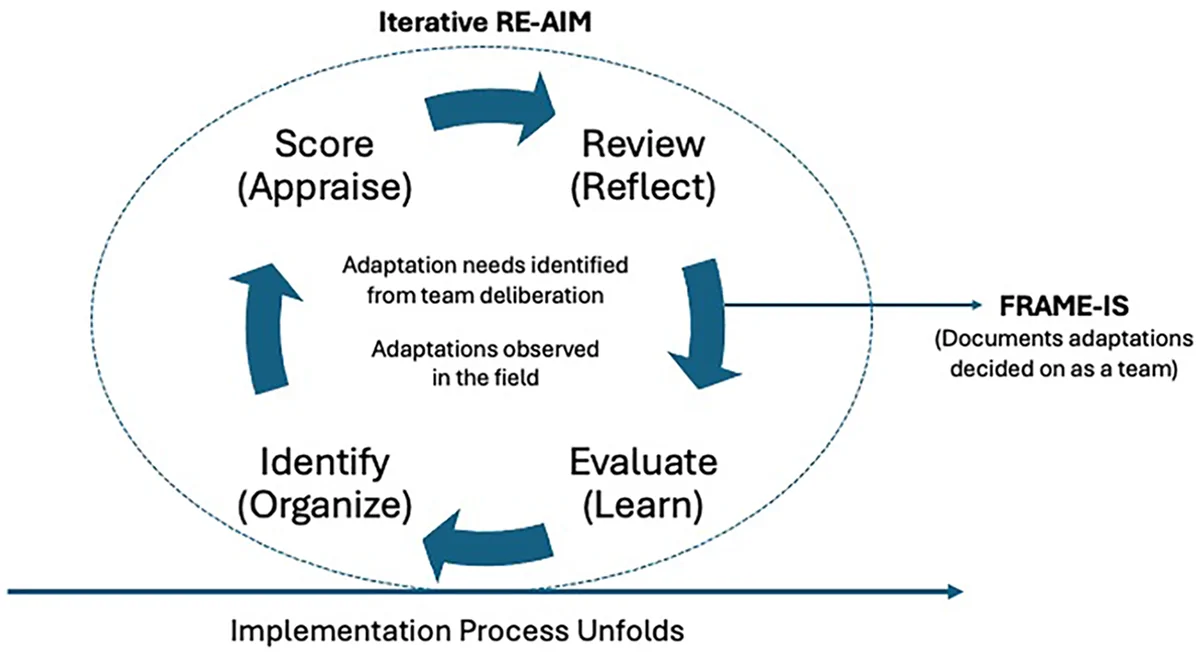
Adaption learning cycle. The adaptation learning cycle integrates Iterative RE-AIM with FRAME-IS to support systematic, ongoing adaptation during implementation. The cycle comprises four sequential and repeating steps, corresponding to the four Iterative RE-AIM steps (Identify, Score, Review, Evaluate): Organize, in which research teams identify participants in the process and organize information on adaptation-related information; Appraise, in which teams solicit input on adaptation needs, observed adaptations, and proposed adaptations; Reflect, in which teams review input, reflect on adaptations, and decide on adaptations; and Learn, in which teams execute selected adaptations, evaluate their effects, and use these results to inform the next cycle. FRAME-IS is used throughout the cycle to document and finalize adaptation decisions arising from this process. The cycle repeats at a cadence determined by each implementation context (e.g., weekly for TEAM EFFORT; once per implementation wave for 40 WINKS).

## Results

### Operationalizing Iterative RE-AIM to support an adaptation learning cycle during implementation

The TEAM EFFORT and 40 WINKS studies operationalized the Iterative RE-AIM approach differently for two primary reasons. First, each study incorporated the Iterative RE-AIM approach at a different point in its implementation timeline. TEAM EFFORT incorporated Iterative RE-AIM principles midway through Wave 1, whereas 40 WINKS adopted the approach near the end of Wave 2. These were the timepoints at which both research teams (and the shared PIs) discovered the need for Iterative RE-AIM and integrated it into each study. Second, differences in study scope, coaching procedures, organizational context, and available personnel resources influenced how each study operationalized the Iterative RE-AIM approach while remaining consistent with underlying intent.

Both studies used the Iterative RE-AIM approach to support ongoing adaptation of both the intervention and implementation strategy. Table 2 compares how each study interpreted and operationalized the four Iterative RE-AIM steps to local implementation needs. Although the operationalizations differed, both studies remained faithful to the underlying intent of the Iterative RE-AIM approach.

### TEAM EFFORT

TEAM EFFORT operationalized the Iterative RE-AIM approach through weekly adaptation learning discussions involving coaches and members of the research team. A research team member facilitated these discussions (Organize). Each week, the group debriefed the previous week's coaching sessions, discussed adaptation needs, reviewed adaptations emerging across nursing homes, considered potential improvements to both the intervention and implementation strategy, and proposed solutions.

Rather than implementing a formal survey/scoring process, the research team used an “input tool” (separate from FRAME-IS) during weekly meetings to collect and collate input. The input tool organized adaptation needs, captured observed adaptations, documented proposed solutions, and prioritized the various adaptations (Appraise). Team members also entered observations into the input tool between meetings whenever new adaptation opportunities emerged during implementation.

Each week, the research team collectively reflected on proposed adaptations, determined which modifications to implement, and incorporated those modifications into the coaching curriculum (Reflect). During subsequent weeks, the team evaluated the effects of implemented adaptations and used those experiences to inform the next round of adaptation learning discussions (Learn).

### 40 WINKS

40 WINKS operationalized the Iterative RE-AIM approach through structured adaptation learning discussions held once during each implementation wave (i.e., every four months). Coaches and research team members participated, with a research team member serving as facilitator (Organize). Similar to TEAM EFFORT, discussions centered on adaptations to both the intervention and implementation strategy as implementation unfolded.

Two weeks before each end-of-wave adaptation learning discussion, participants completed a structured survey (Appraise). The survey asked participants to evaluate adaptations implemented during the current wave, assess understanding of the coaching curriculum and intervention, provide open-ended feedback regarding the current implementation experience, and propose new adaptations.

Survey results were summarized and shared before the adaptation learning discussion. During the two-hour meeting, participants reviewed survey findings, reflected on adaptations implemented during the current wave, brainstormed additional adaptations, and collectively prioritized adaptations for the subsequent wave (Reflect). Adaptations selected to be implemented during the following wave were evaluated during the next adaptation learning discussion (Learn).

This operationalization reflected the longer implementation timeline, broader organizational scope, and larger collaborative structure of the 40 WINKS trial. Rather than embedding adaptation learning into weekly meetings, the study intentionally consolidated learning across an entire implementation wave before making coordinated decisions regarding future intervention and implementation strategy adaptations. However, participants were encouraged and supported to gather information about adaptations between meetings. For example, the coaching team met weekly with two research team members and was regularly encouraged to share observations and evaluations to be included in the meeting minutes to inform the adaptation learning discussion (Organize, Learn).

## Discussion

This paper describes how the TEAM EFFORT and 40 WINKS trials paired Iterative RE-AIM with FRAME-IS to enable an adaptation learning cycle. Below, we reflect on how each trial operationalized the Iterative RE-AIM protocol written by Glasgow to the specific setting, context, and needs of each trial, while remaining faithful to Iterative RE-AIM's underlying intent through the processes of Organize, Appraise, Reflect, and Learn. We also reflect on how Iterative RE-AIM strengthened implementation, explain the rationale underlying our operationalization decisions, and describe changes we would make in future applications.

### Organize

Teams from both trials felt that Iterative RE-AIM fostered inclusive participation of team members and reduced siloing that could have hindered adaptations. Inclusivity is a central LOCK principle, so inclusive decision-making was reflected in both the coaching implementation strategy and the Iterative RE-AIM process. Just as coaches encouraged nursing homes to incorporate input from staff of all levels (e.g., certified nursing assistant, custodial staff) into decisions about resident care, the research team sought to include additional voices (e.g., coaches) into the Iterative RE-AIM process and Adaptation Learning Team, empowering all team members to lend their voices to the adaptation process. In the future, we would seek to further our inclusivity by soliciting more input and representation from the nursing homes (e.g., leadership and champion teams, direct care staff, management companies).

Moreover, we decided to center our Iterative RE-AIM discussions around adaptations to the intervention (LOCK huddle) and the implementation strategy (coaching curriculum and processes used by coaches), not to RE-AIM outcomes. First, we centered discussions around adaptations because we felt implementing the adaptations would pose the greatest challenge. So, spending time discussing adaptations would better help the trial make timely progress. Second, because adaptations and modifications are inherently intended to improve RE-AIM outcomes, discussing the intervention and implementation strategy became the practical mechanism to advance RE-AIM domains. The study design and plans for measurement/evaluation were already explicitly grounded in RE-AIM. So we felt like RE-AIM domains were still being implicitly discussed and thus felt the Iterative RE-AIM spirit was preserved.

### Appraise

Our trials show how “Appraise” can be operationalized by varying both the methods of data collection (e.g., survey vs. custom input tool) and the level at which input is collected (e.g., individual vs. group-level). 40 WINKS used an individual-level survey, whereas TEAM EFFORT used a group-level input tool. The essence of “Appraise” is to actively solicit and collect input about adaptations needs across timepoints (e.g., prior wave, current wave) and from different sources (e.g., research team vs. coaches vs. community nursing home members).

40 WINKS collected individual-level input through a survey, consistent with the approach suggested by Glasgow. The survey cued team members to individually reflect and consolidate what the team had learned over several months. This laid the groundwork for deeper level of reflection, discussion, and adaptation development during the adaptation learning team gathering and enabled the team to think more holistically and prospectively (e.g., “I want to bring this up so we can improve this process for the next wave.”).

TEAM EFFORT collected group-level input through a custom input tool (separate from FRAME-IS) used during weekly meetings. The input tool was distinct from, but complementary to, FRAME-IS in both its scope and purpose. FRAME-IS documented finalized adaptation decisions, including what adaptations were made and why. In contrast, the input tool functioned as an earlier brainstorming partner, capturing information and ideas that had not yet matured into decisions, including adaptation needs, observed adaptations that emerged naturally from the field, and proposals for new adaptations.

These two approaches to Appraise each have advantages. TEAM EFFORT demonstrates how appraisal can be embedded efficiently within routine team meetings, allowing adaptation-related information to be captured and considered as implementation unfolds. The 40 WINKS survey created an opportunity for individual team members to digest and consolidate their observations and contribute their perspectives before collective reflection. In future applications, when resources permit, researchers might consider combining these approaches: ongoing group-level appraisal throughout implementation supplemented by periodic individual-level assessment and structured team reflection. This combined approach could preserve the immediacy and efficiency of TEAM EFFORT while extending the breadth of participation and depth of reflection afforded by 40 WINKS.

### Reflect

One of the core principles of Iterative RE-AIM is giving teams the space not only to reflect, but to reflect *together*. After “Appraise”– where pieces of information are solicited, collected, and organized—“Reflect” prompts everyone to synthesize the information together and jointly agree upon a path forward.

For 40 WINKS, the “Appraise” step occurred at the individual level (survey), followed by a “Reflect” step occurring at a larger evaluation meeting as a team. In contrast, TEAM EFFORT demonstrated how “Appraise” and “Reflect” could coincide concurrently. Carrying out “Appraise” and “Reflect” concurrently was the most feasible choice because of the smaller size of the core research team and fewer available resources. In this way, “Appraise” and “Reflect” always occurred at the “team level” during the weekly team meetings.

By prompting our research teams to systematically solicit input (either through a formal survey or weekly meetings), Iterative RE-AIM enabled each study to identify implementation challenges and promote adaptation development in real-time. The rapid speed at which complex, multi-site clinical trials advance makes it challenging for research teams to nimbly respond to participants’ needs. But Iterative Re-AIM helped our trials respond to participants’ needs in a timely fashion.

### Learn

The two studies differed in how frequently to learn and repeat the adaptation learning cycle. TEAM EFFORT met weekly for shorter meetings, and 40 WINKS met once-a-wave for a longer evaluation meeting. Given their resources and timeline, TEAM EFFORT felt a weekly frequency was an appropriate cadence to match the ever-changing needs of its sites in a compressed design (the trial included just two waves). Participating nursing homes experienced a variety of challenges during the implementation period (e.g., leadership changes, outbreaks of influenza and norovirus), so a weekly cadence was the most appropriate response to these challenges. 40 WINKS decided on once-a-wave because they had more phases in the study and a longer timeline. A once-a-wave cadence was felt to be appropriate and the study team focused these once-a-wave reflections on digesting the lessons learned from one wave to prepare for the next. Ultimately, our presentation of both approaches underscores that there is no single “correct” frequency for evaluation; rather, the cadence should be determined by the pace and complexity of the implementation context itself. Flexibility in this step ensured that adaptation remained a living, responsive process rather than a procedural checkbox.

## Conclusions

Pairing Iterative RE-AIM with FRAME-IS offers a systematic approach to adaptation learning during implementation, combining systematic documentation of adaptations with structured team deliberation. The TEAM EFFORT and 40 WINKS trials demonstrate that Iterative RE-AIM can be operationalized in substantially different ways while remaining faithful to its underlying intent. Rather than prescribing a single implementation protocol, our findings suggest that decisions about how to Organize, Appraise, Reflect, and Learn can, and should, respond to the setting, resources, team structure, and pace of implementation. We hope this narrative account encourages researchers to approach Iterative RE-AIM as a flexible, principled process for enabling adaptation learning in complex implementation environments. Future work should test these customization approaches across a broader range of populations, interventions, and settings — including assisted living, residential care homes, and beyond long-term care entirely — and should explore the meaningful inclusion of frontline stakeholders, such as direct care staff and residents, in the Iterative RE-AIM process itself.

## Data Availability

The original contributions presented in the study are included in the article/Supplementary Material, further inquiries can be directed to the corresponding author.
